# Immunotherapy for patients with advanced pancreatic carcinoma: a promising treatment

**DOI:** 10.18632/oncotarget.13968

**Published:** 2016-12-15

**Authors:** Bin Zhang, Yuhao Dong, Jing Liu, Zhouyang Lian, Long Liang, Wenbo Chen, Xiaoning Luo, Shufang Pei, Xiaokai Mo, Lu Zhang, Wenhui Huang, Fusheng Ouyang, Baoliang Guo, Changhong Liang, Shuixing Zhang

**Affiliations:** ^1^ Department of Radiology, Guangdong General Hospital/Guangdong Academy of Medical Sciences, Guangzhou, Guangdong, P.R. China; ^2^ Graduate College, Southern Medical University, Guangzhou, Guangdong, P.R. China; ^3^ Department of Radiology, Huizhou Municipal Central Hospital, Huizhou, Guangdong, P.R. China; ^4^ School of medicine, South China University of Technology, Guangzhou, Guangdong, P.R. China

**Keywords:** immunotherapy, chemotherapy, advanced pancreatic cancer, adverse events, overall survival

## Abstract

There are limited data on the safety and efficacy of immunotherapy for patients with advanced pancreatic cancer (APC). A meta-analysis of single-arm trials is proposed to assess the efficacy and safety of immunotherapy for APC. Eighteen relevant studies involving 527 patients were identified. The pooled disease control rate (DCR), overall survival (OS), progression free survival (PFS), and 1-year survival rate were estimated as 59.32%, 7.90 months, 4.25 months, and 30.12%, respectively. Subgroup analysis showed that the pooled OS, PFS, and 1-year survival rate were significantly higher for autologous activated lymphocyte therapy compared with peptide-based vaccine therapy (OS: 8.28 months vs. 7.40 months; PFS: 6.04 months vs. 3.86 months; 1-year survival rate: 37.17% vs. 19.74%). Another subgroup analysis demonstrated that the pooled endpoints were estimated as obviously higher for immunotherapy plus chemotherapy compared with immunotherapy alone (DCR: 62.51% vs. 47.63%; OS: 8.67 months vs. 4.91 months; PFS: 4.91 months vs. 3.34 months; 1-year survival rate: 32.32% vs. 21.43%). Of the included trials, seven trials reported no treatment related adverse events , five trials reported (16.6 3.9) % grade 3 adverse events and no grade 4 adverse events. In conclusion, immunotherapy is safe and effective in the treatment of APC.

## INTRODUCTION

Pancreatic cancer (PC) is the fourth most common cause of cancer death worldwide, which is characterized by an extremely poor survival rate [1]. Up to 80% deaths occur

within the first year of diagnosis and the overall 5-year mortality rate is over 95% [2, 3]. Although surgical resection is the only potentially curative approach, only 10-20% of pancreatic tumors are operable, about 40% are locally advanced, unresectable and 45% are with metastases [4, 5]. Usually, patients with advanced pancreatic cancer (APC) are unable to resort to surgery, the treatment alternatives of whom are very limited, with gemcitabine as the current first-line treatment [6]. However, patients received gemcitabine had a overall survival (OS) of around 6 months and a one-year survival ≤ 20% [7]. Once APC patients are resistant to gemcitabine therapy, there is barely effective treatment. New treatment strategies are therefore urgently required.

Recently, immune cell-based cancer therapy has been attempted as an alternative treatment option for anticancer therapy [8]. It eliminates cancer cells by modulating the immune system to suppress cancer by active (potentiating the patient's intrinsic immune system against cancer cells ) and passive (administering extrinsic man-made immune system components ) immunotherapy [9]. Immunotherapy has an advantage over chemo(radio) therapies due to its specificity against tumor without hurting normal tissue [10]. Immunotherapeutic methods to PC included the administration of antibodies [11], cytokines [12], peptide vaccines [6,13–18], and autologous activated lymphocyte (e.g. dendritic cells, lymphokine activated killer cells, and cytotoxic T-lymphocyte) therapies [10, 19–24]. Despite the limited benefit of gemcitabine, it may enhance responses to specific vaccines or synergize immune system stimulators [22]. Hence, combining gemcitabine with immunotherapy for APC patients was used in most clinical trials. However, these published trials have been small with consistent results, precluding robust estimates of benefit.

To assess the benefit of immunotherapy in the treatment of patients with APC neutrally, we would like to perform a published data meta-analysis of all relevant single-arm trials.

## RESULTS

### Trials

**Figure 1:**
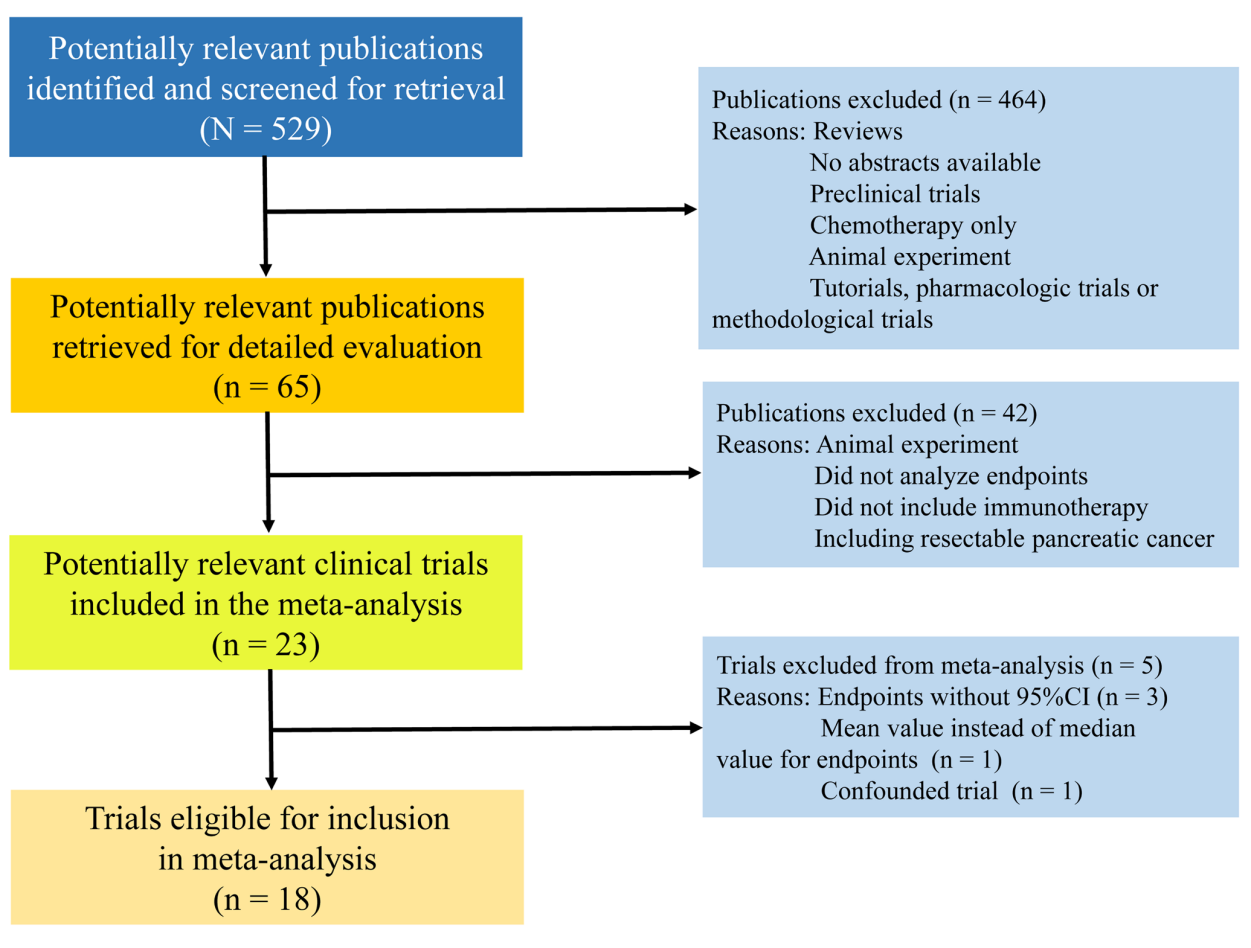
Study flowchart

A total of 18 trials involving 527 patients met the inclusion criteria were enrolled in this study (Figure 1). It included seven trials (295 patients) of autologous activated lymphocyte therapies, eight trials (170 patients) of peptide-based vaccine therapy, one trial (34 patients) of monoclonal antibody plus gemcitabine, one trial (16 patients) of cytokine-induced killer (CIK) and one trial (12 patients) of gene-mediated cytotoxic immunotherapy (GMCI). There were 14 trials (454 patients) used immunotherapy plus chemotherapy, only four trials (73 patients) used immunotherapy alone. Table 1 shows the details of trial designs, publication year, number of patients, female/male ratio, treatment schedules, and study end points.

**Table 1:** Detailed data of the 18 trials included in this meta-analysis

Trial	study design	No. of patients	F/M ratio	Age	Treatment protocol	Study endpoints
KONDO (2008)	NA	20	6/14	63.4	MUC1-DC+CTL	DCR, OS, PFS, 1-year survival rate, toxicity
Shindo (2014)	Phase II	42	21/21	63.1	MUC1-DC+CTL+ GEM	DCR,MST, 1-year survival rate, , toxicity
Hirooka (2009)	Phase I	5	4/1	57.2	DC+CTL+GEM	DCR,PFS,OS,1-year survival rate, toxicity
Gansauge (2013)	Phase I/II	134	58/76	63.9	LANEX-DC+GEM	DCR, OS, 1-year survival rate, toxicity
Kimura (2011)	Phase I	49	7/42	61.7	DC+LAK+GEM/S-1	DCR, OS, 1-year survival rate, toxicity
Aglietta (2014)	Phase I	34	12/22	59.5	Tremelimumab +GEM	OS, toxicity
Aguilar (2015)	Phase I	12	NA	65.6	GMCI+chemoradiation	DCR, OS, PFS, 1-year survival rate, toxicity
Asahara (2013)	Phase I	31	14/17	61.3	HLA-A24 peptide vaccine	DCR, PFS, OS,1-year survival rate, toxicity
Kameshima (2013)	NA	6	3/3	61.2	Survivin-2B80-88 peptide	DCR
Chung (2014)	Phase II	16	NA	59.5	CIK cells	DCR, OS, PFS, 1-year survival rate, QoL, toxicity
Suzuki (2014)	Phase I	9	5/4	61.8	KIF20A+GEM	DCR,PFS,OS,1-year survival rate, toxicity
Nishida (2014)	Phase I	31	15/17	60.0	WT1 peptide+GEM	DCR,PFS,OS,1-year survival rate, toxicity
STAFF (2014)	Phase III	21	7/14	66.1	GV1001+GEM	DCR,PFS,OS,1-year survival rate, toxicity
Miyazawa (2010)	Phase I	18	4/14	65.3	VEGFR2-169+GEM	DCR, OS, PFS, toxicity
YUTANI (2013)	Phase II	41	14/27	61.0	Personalized peptide vaccines	DCR, MST, 1-year survival rate, toxicity
Yanagimoto (2007)	Phase I	13	4/9	62.3	Personalized peptide vaccines	DCR, OS, PFS, 1-year survival rate, toxicity
NAKAMURA (2009)	Phase I/II	17	10/7	63.0	DC ± LAK +GEM	OS, 1-year survival rate
KANEKO (2005)	Phase II	28	9/17	63.9	DC/LAK+GEM	DCR, OS, toxicity

Note: F/M = Female/Male NA = Not Available DC = Dendritic cells CTL = cytotoxic T-lymphocyte LAK =

lymphokine-activated killer lymphocytes GEM = Gemcitabine CIK = Cytokine-induced killer DCR = Disease Control Rate PFS = Progression-Free Survival OS = Overall Survival QoL = Quality of Life WT1 = Wilms Tumor Gene

### Disease control rate

**Figure 2:**
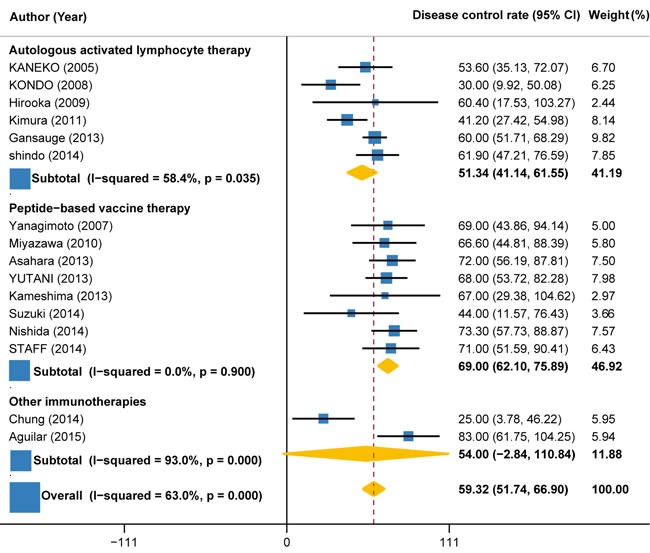
Disease control rate in trials of autologous activated lymphocyte therapy versus peptide-based vaccine therapy versus other therapy

**Figure 3:**
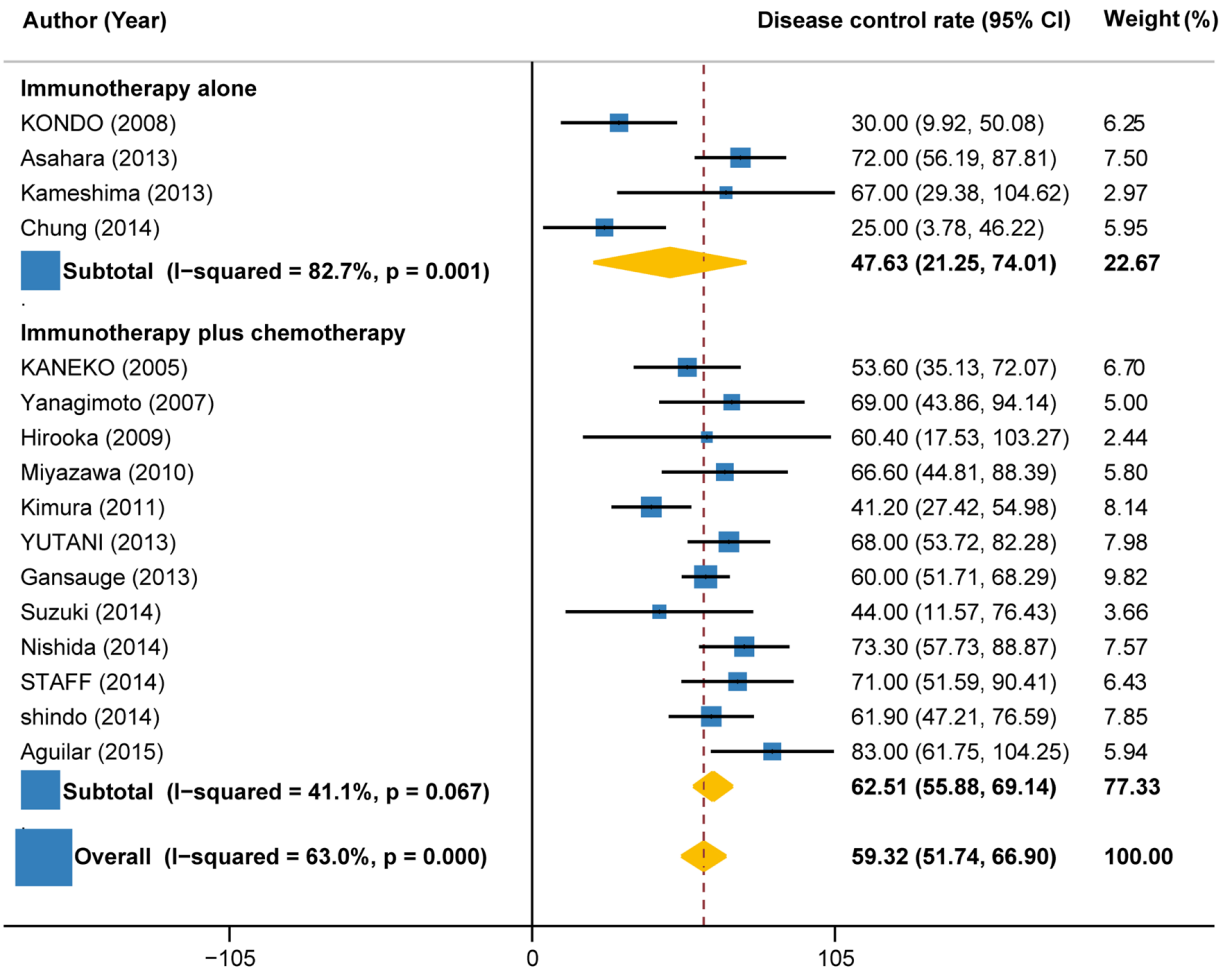
Disease control rate in trials of immunotherapy versus immunotherapy plus chemotherapy

Disease control rate, defined as additive rates of complete response, partial response and stable disease, which could be calculated from all trials but two. Response classification was based on the Response Evaluation Criteria in Solid Tumors (RECIST). A total of 476 (90%) patients’ response data were available, of which, 290 (61%) patients received response. The pooled DCR for immunotherapy and/or chemotherapy was estimated as 59.32% (95%CI: 51.74% to 66.90%) (Figure 2). However, significant heterogeneity was observed between the trials (I2 = 63%, P < 0.001). Subgroup analysis by the type of immunotherapy showed that the DCR of peptide-based vaccine therapy (69.0%, 95%CI: 62.10% to 75.89%; I2 = 0%, P = 0.900) increased by more than 34% as compared to autologous activated lymphocyte therapy (51.34%, 95%CI: 41.14% to 61.55%; I2 = 58.4%, P = 0.035) (Figure 2). Another subgroup analysis by the combination with or without chemotherapy indicated that the DCR for immunotherapy plus chemotherapy (62.51%, 55.88% to 69.14%; I2 = 41.4%, P = 0.067) was 31% higher than immunotherapy alone (47.63%, 95%CI: 21.25% to 74.01%; I2 = 82.7%, P = 0.001) (Figure 3).

### Progression free survival

**Figure 4:**
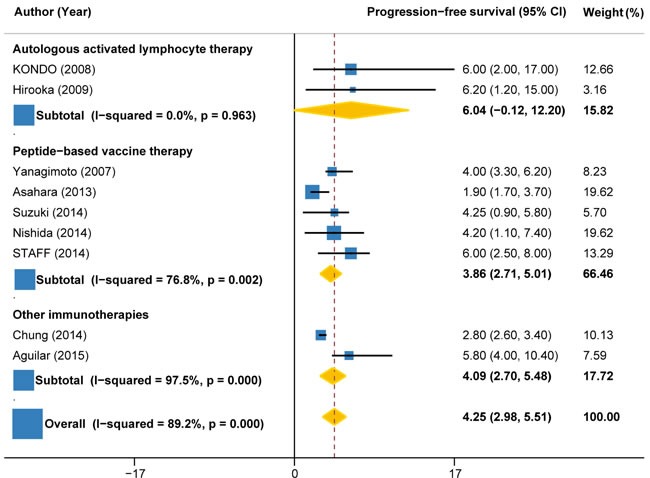
Progression-free survival in trials of autologous activated lymphocyte therapy versus peptide-based vaccine therapy versus other therapy

**Figure 5:**
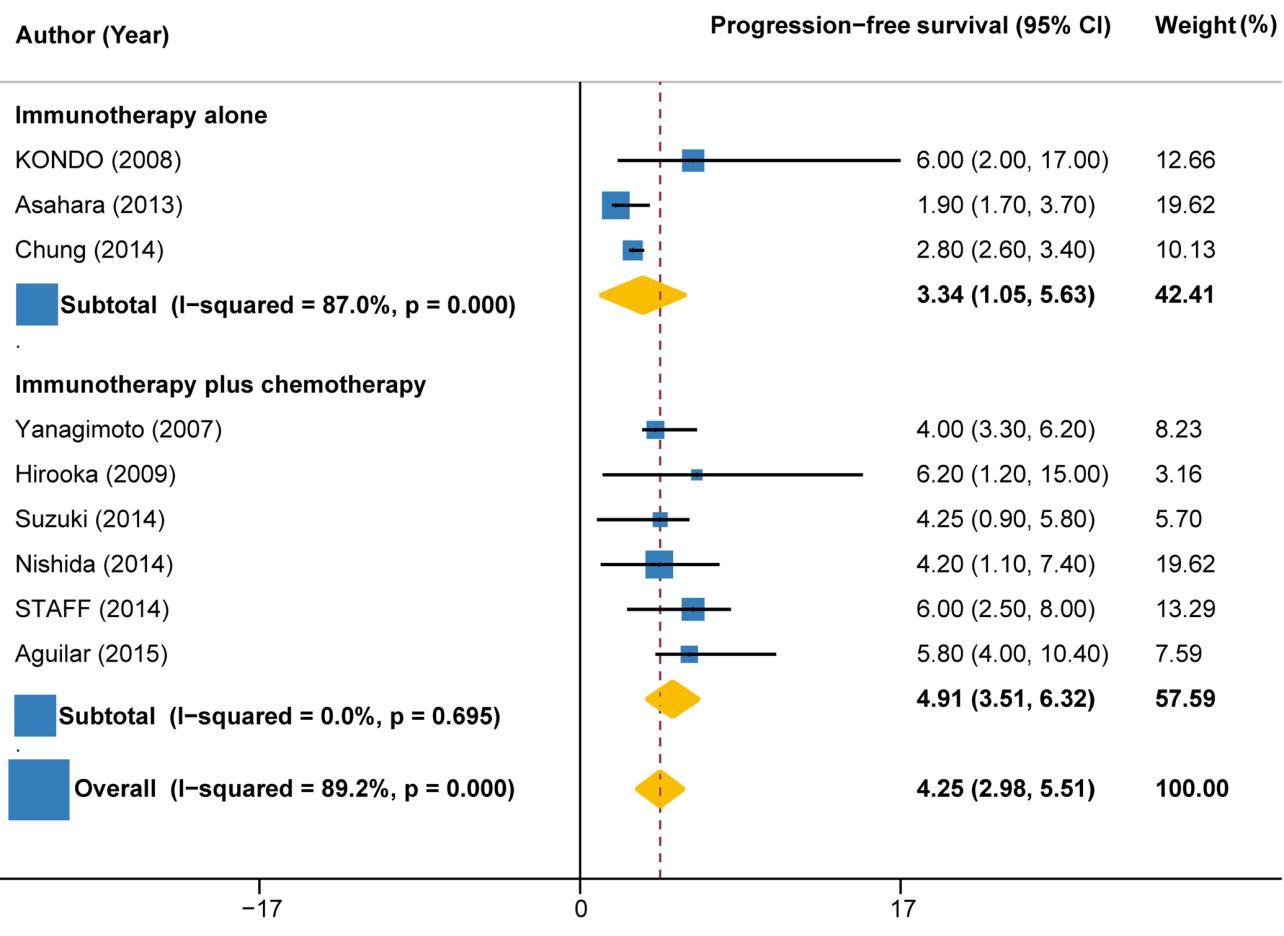
Progression-free survival in trials of immunotherapy versus immunotherapy plus chemotherapy

Progression-free survival (PFS) time was reported in nine trials (158 patients). The pooled PFS for immunotherapy was estimated to be 4.25 months (95%CI: 2.98 to 5.51), but with significant heterogeneity between individual trials (I2 = 89.2%, P < 0.001) (Figure 4). Subgroup analysis by the type of immunotherapy showed that the PFS was significant longer for autologous activated lymphocyte therapy (6.04 months, 95%CI: -0.12 to 12.20; I2 = 0%, P = 0.963) than peptide-based vaccine therapy (3.86 months, 95%CI: 2.71 to 5.01; I2 = 76.8%, P = 0.002) (Figure 4). Subgroup analysis according to the combination with or without chemotherapy indicated that the PFS was much longer for immunotherapy combined with chemotherapy (4.91 months, 95%CI: 3.51 to 6.32; I2 = 0%, P = 0.695) compared with immunotherapy alone (3.34 months, 95%CI: 1.05 to 5.63; I2 = 87%, P < 0.001) (Figure 5).

### Overall survival

**Figure 6:**
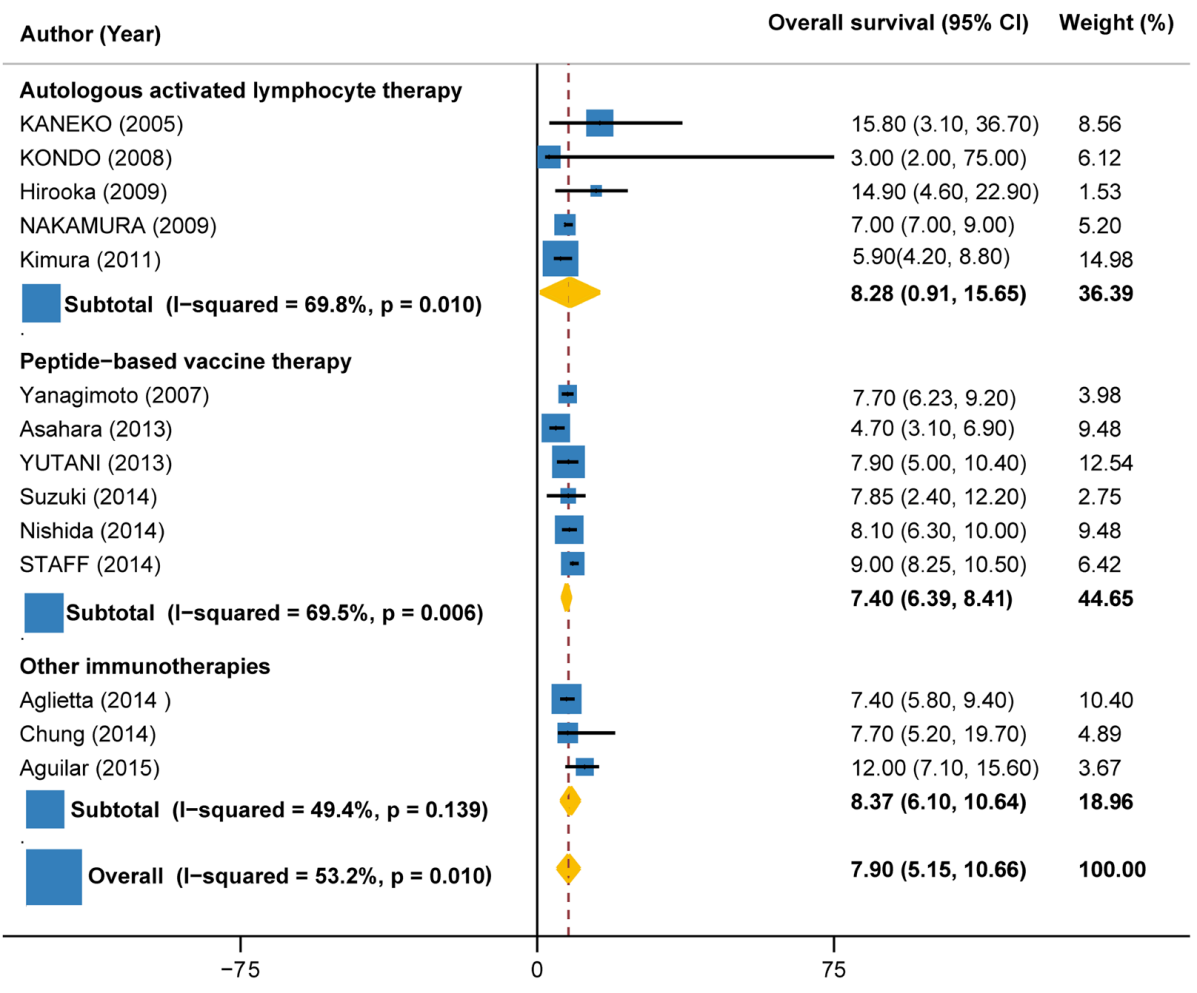
Overall survival in trials of autologous activated lymphocyte therapy versus peptide-based vaccine therapy versus other therapy

**Figure 7:**
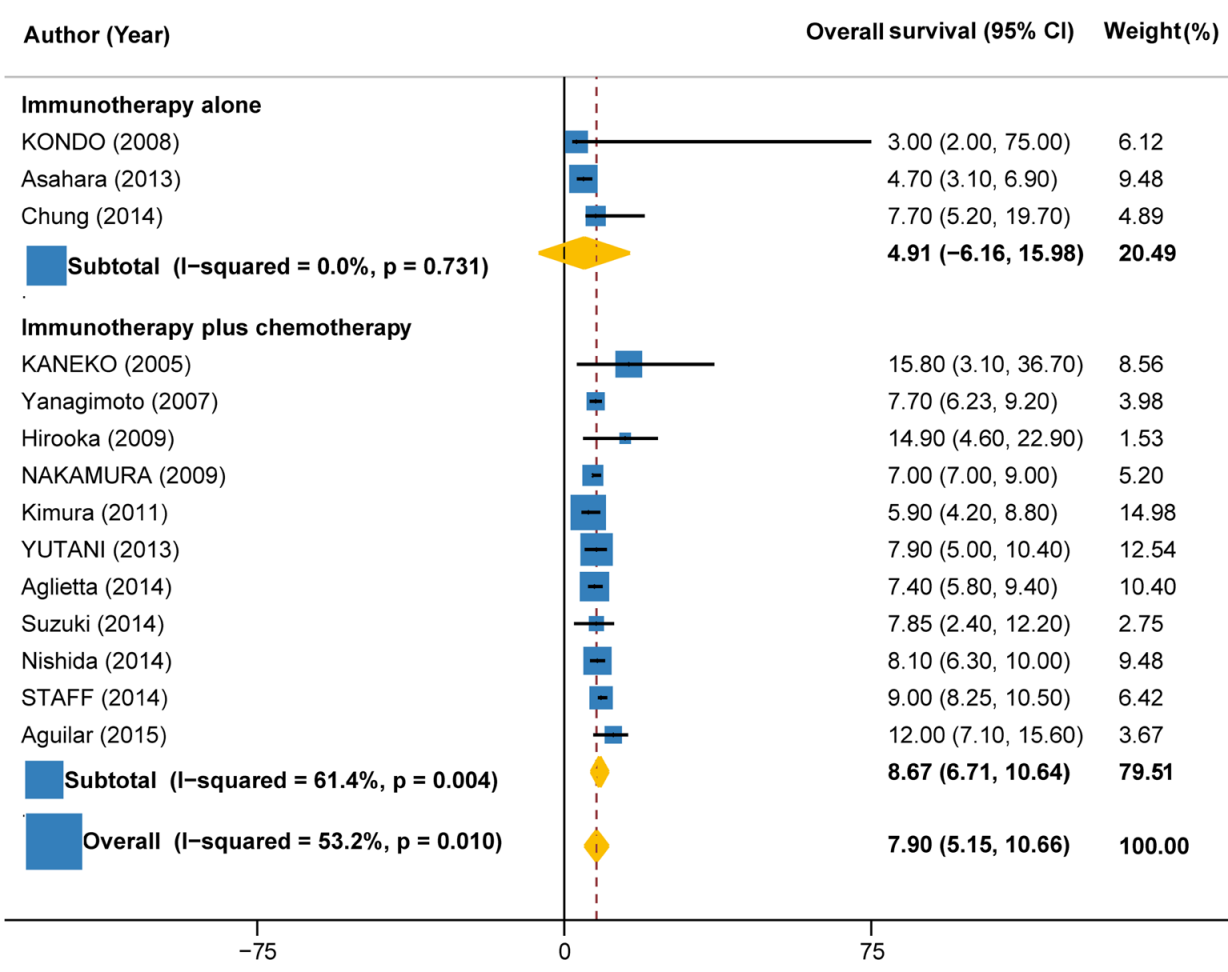
Overall survival in trials of immunotherapy versus immunotherapy plus chemotherapy

Overall survival data were available in 14 trials (327 patients), with values ranging from 3 months to 14.9 months. Overall, the pooled OS for immunotherapy was estimated as 7.90 months (95%CI: 5.15 to 10.66), but with significant heterogeneity between individual trials (I2 = 53.2%, P = 0.010) (Figure 6). Subgroup analysis by the type of immunotherapy showed that the benefit was more significant for autologous activated lymphocyte therapy (8.28 months, 95%CI: 0.91 to 15.65; I2 = 69.8%, P = 0.010) compared with peptide-based vaccine therapy (7.40 months, 95%CI: 6.39 to 8.41; I2 = 69.5%, P = 0.006) (Figure 6). Subgroup analysis by the combination with or without chemotherapy demonstrated that the benefit was more significant for immunotherapy plus chemotherapy (8.67 months, 95%CI: 6.71 to 10.64; I2 = 61.4%, P = 0.004) as compared with immunotherapy alone (4.91 months, 95%CI: -6.16 to 15.98; I2 = 0%, P = 0.731) (Figure 7).

### 1-year survival rate

**Figure 8:**
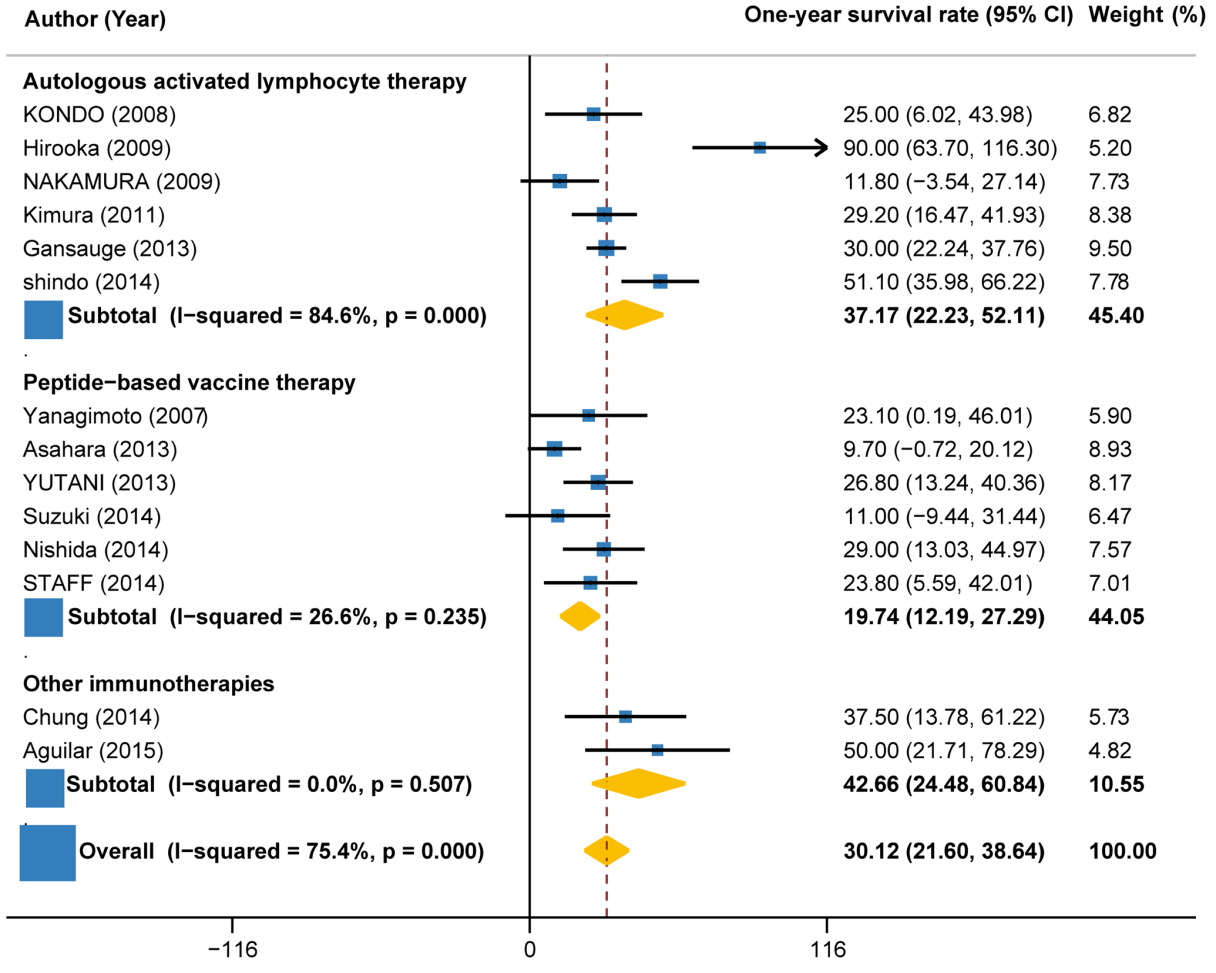
1-year survival rate in trials of autologous activated lymphocyte therapy versus peptide-based vaccine therapy versus other therapy

**Figure 9:**
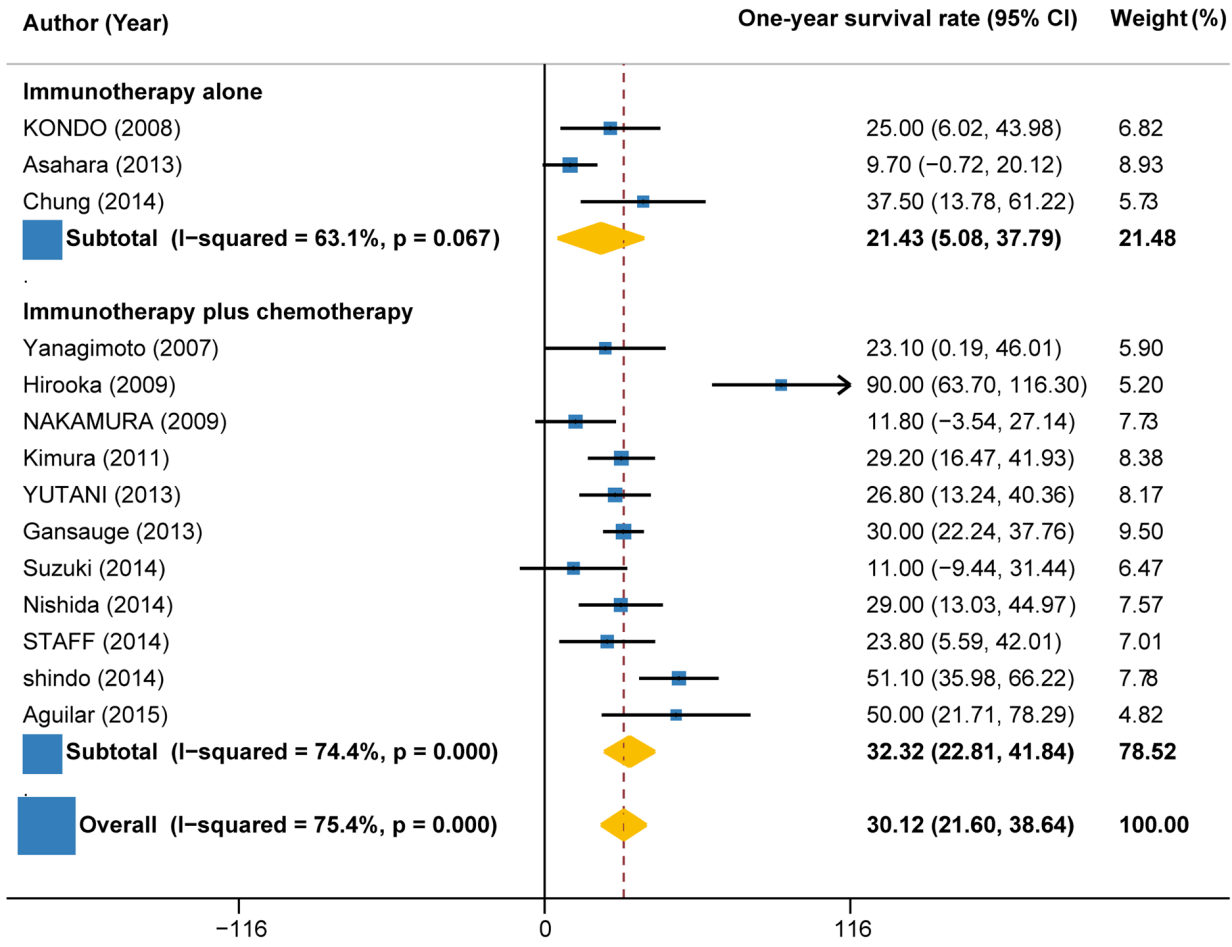
1-year survival rate in trials of immunotherapy versus immunotherapy plus chemotherapy

1-year survival rate could be calculated from 14 trials (441 patients). The pooled 1-year survival rate for immunotherapy ± chemotherapy was estimated as 30.12% (95%CI: 21.60% to 38.64%; I2 = 75.4%, P < 0.001) (Figure 8). Subgroup analysis by the type of immunotherapy demonstrated that the 1-year survival rate for autologous activated lymphocyte therapy (37.17%, 95%CI: 22.23% to 52.11%; I2 = 84.6%, P < 0.001) was 88% higher than that of peptide-based vaccine therapy (19.74%, 95%CI: 12.19% to 27.29%; I2 = 26.6%, P = 0.235) (Figure 8). Subgroup analysis by the combination with or without chemotherapy showed that the 1-year survival rate for immunotherapy + chemotherapy (32.32%, 22.81% to 41.84%; I2 = 74.4%, P < 0.001) was about 51% higher than that of immunotherapy alone (21.43%, 95%CI: 5.08% to 37.79%; I2 = 63.1%, P = 0.067) (Figure 9).

### Treatment-related adverse events

Adverse events were monitored based on the National Institute Common Terminology Criteria for Adverse Events version 3.0. Reports on treatment-related adverse events (TRAEs) were variable among trials. Of the 18 trials, 9 trials (7 using autologous activated lymphocyte therapy ± gemcitabine, 1 using peptide-based vaccine therapy, and 1 using monoclonal antibody + gemcitabine) reported no TRAEs, 7 trials (5 using peptide-based vaccine therapies, 1 using gene-mediated cytotoxic immunotherapy, and 1 using cytokine-induced killer cells) reported grade 3 TRAEs (e.g. anemia, lymphopenia, leukopenia, neutropenia, thrombocytopenia) with mean rate of 14.56% (ranging from 3.22% to 25.8%) and no grade 4 TRAEs. The remaining 2 trials did not report TRAEs.

### Meta-regression analyses

No variables met statistical significance on meta-regression analyses (P > 0.05 for all) (Tables 2, 3, 4, 5).

**Table 2:** Univariate meta-regression analysis of possible sources of heterogeneity across the included trials reporting OS

Possible source of heterogeneity	Trials, n	Co-efficient (95%CI)	P value
Study design	13	Phase I: -2.10 (-23.27, 19.07) Phase I/II: -2 (-27.79, 23.79) Phase II: -0.33 (-23.85, 23.19)	0.827 0.865 0.975
Number of patients	14	-0.01 (-0.36, 0.33)	0.941
Female/Male ratio	13	0.027 (-0.051, 0.105)	0.459
Publication year	14	0.43 (-094, 1.79)	0.509
Patients’ age	14	-0.396 (-2.609, 1.817)	0.703
Type of immunotherapy	14	AALT: -3.68 (-15.67, 8.31) PBVT: -1.47 (-13.91, 10.98)	0.513 0.800
Combination ± chemotherapy	14	4.12 (-3.44, 11.68)	0.258

**Table 3:** Univariate meta-regression analysis of possible sources of heterogeneity across the included trials reporting PFS

Possible source of heterogeneity	Trials, n	Co-efficient (95%CI)	P value
Study design	8	Phase I: -2.88 (-18.72, 12.96) Phase II: -3.2 (-20.22, 13.82)	0.660 0.649
Number of patients	9	-0.105 (-0.420, 0.209)	0.453
Female/Male ratio	8	-0.007 (-0.038, 0.527)	0.710
Publication year	9	-0.19 (-1.43, 1.06)	0.732
Patients’ age	9	0.28 (-1.27, 1.83)	0.686
Type of immunotherapy	9	AALT: -2.73 (-9.49, 14.95) PBVT: -0.41 (-7.52, 6.70)	0.604 0.892
Combination ± chemotherapy	9	-2.31 (-8.20, 3.57)	0.383

**Table 4:** Univariate meta-regression analysis of possible sources of heterogeneity across the included trials reporting DCR

Possible source of heterogeneity	Trials, n	Co-efficient (95%CI)	P value
Study design	14	Phase I: -15.9 (-180.4, 148.6) Phase I/II: -11 (-218.1, 196.1) Phase II: -34.1 (-87.2, 229.2)	0.834 0.908 0.654
Number of patients	16	-0.156 (-0.872, 1.185)	0.749
Female/Male ratio	15	0.037 (-0.333, 0.407)	0.832
Publication year	16	-0.06 (-0.907, 8.95)	0.989
Patients’ age	16	3.05 (-8.84, 14.93)	0.591
Type of immunotherapy	16	AALT: 14.09 (-551.53, 79.7) PBVT: 30.72 (-39.43, 100.87)	0.650 0.361
Combination ± chemotherapy	16	-23.9 (-76.5, 28.7)	0.346

**Table 5:** Univariate meta-regression analysis of possible sources of heterogeneity across the included trials reporting 1-year survival rate

Possible source of heterogeneity	Trials, n	Co-efficient (95%CI)	P value
Study design	13	Phase I: -9.55 (-65.36, 46.27) Phase I/II: -9.56 (-68.86, 49.73) Phase II: 9.80 (-60.6, 80.21)	0.708 0.724 0.760
Number of patients	14	0.131 (-0.38, 0.65)	0.590
Female/Male ratio	14	-0.003 (-0.311, 0.65)	0.590
Publication year	14	-0.189 (-4.99, 4.62)	0.913
Patients’ age	14	-0.322 (-6.939, 6.296)	0.917
Type of immunotherapy	14	AALT: -22.73 (-91.86, 46.40) PBVT: -28.02 (-95.49, 39.46)	0.484 0.380
Combination ± chemotherapy	14	5.02 (-18.59, 28.64)	0.651

### Publication bias

Both the Egger's and Begg's tests revealed no publication bias for OS (P = 0.951 and 0.956, respectively) and for PFS (P = 0.085 and 0.754, respectively). Begg's test not Egger's test revealed no publication bias for 1-year survival rate (P = 0.815 and 0.000, respectively). Both the Egger's and Begg's tests revealed publication bias for DCR (P = 0.017 and 0.000, respectively).

## DISCUSSION

The present meta-analysis gathers all currently available data from single-arm studies reporting immunotherapy for those patients with advanced pancreatic cancer. To our best of knowledge, ours is the first meta-analysis to analyze the efficacy and safety of immunotherapy using single-arm trials. The results showed immunotherapy is more effective than other previously reported standard treatmentin terms of OS, PFS, 1-year survival rate for advanced pancreatic carcinoma [28–32]. After stratifying trials according to the type of immunotherapy, we found that the benefits of autologous activated lymphocyte therapy were more significant than that of peptide-based vaccine therapy (OS: 8.28 months vs. 7.40 months; PFS: 6.04 months vs. 3.86 months; 1-year survival rate: 37.17% vs. 19.74%). Since the early 1990s, many institutions have actively studied the use of autologous activated lymphocyte therapies, including lymphokine-activated killer lymphocytes (LAK), cytotoxic T-lymphocyte (CTL), and dendritic cell (DC) [33]. Among various immune cell types studied as potential candidates for effective immunotherapy, DCs are potent antigen-presenting cells that participate in the initiation of T-cell immunity and involved in the regulation of both innate and adoptive immune response [24, 34]. Some previous clinical trials have used the combination antigen-pulsed or peptide-pulsed DC vaccine with LAK cells or CTLs in patients with advanced pancreatic cancer and observed significantly prolonged survival time [10, 20, 21, 23, 24].

As a matter of fact, all trials performed so far comparing immunotherapy with standard therapies failed to show the superiority of the former [35]. Obviously, cancer immunotherapy would not be able to take the place of chemotherapy and radiotherapy. Moreover, though recent clinical trials have investigated the sequential administration of immunotherapy and chemo (radio) therapy, the immunosuppressive effects of these standard therapies may weaken the efficacy of immunotherapy. Therefore, it will be essential to find an optimal way to integrate immunotherapy with chemo(radio) therapy. Subgroup analysis indicated that the benefits of combined therapy were more significant than that of immunotherapy alone (DCR: 62.51% vs. 47.63%; OS: 8.67 months vs. 4.91 months; PFS: 4.91 months vs. 3.34 months; 1-year survival rate: 32.32% vs. 21.43%). For chemotherapy, gemcitabine is currently one of the standard therapies for advanced pancreatic cancer, although many chemotherapeutic drugs have been used in clinical trials over the past two decades [35]. However, the effect of gemcitabine is limited and most patients received gemcitabine die within 6 months. Interestingly, some recent reports showed that gemcitabine may improve patients’ response to immunotherapy [22]. Dauer et al have demonstrated in their in vitro study that human pancreatic cancer cell lines could be sensitized by gemcitabine against CTL-mediated lysis [36]. DC vaccine plus gemcitabine prolongs survival time in a animal model [37]. Although some trials of combined therapies including not only gemcitabine but also other cytotoxic drugs showed improved response rates compared with gemcitabine alone, they failed to gain survival benefits [38–41]. Thus, most trials included in this meta-analysis combined only gemcitabine with immunotherapy.

The importance of treatment-related adverse events (TRAEs) that related to immunotherapy must be emphasized. Reporting on TRAEs was variable across trials. Of the included 18 trials, no trails observed related to autologous activated lymphocyte therapy. Five trials using peptide-based vaccine therapies reported (16.6 ± 3.9)% grade III TRAEs and no grade IV TRAEs and one trial reported no TRAEs. Therefore, immunotherapy used in advanced pancreatic cancer patients is safe and no evidence of autoimmune disease was noted, especially for autologous activated lymphocyte therapy.

In summary, cancer immunotherapy was well tolerated and effective in the treatment of advanced pancreatic carcinoma. Currently, available clinical evidences suggest that the combination immunotherapy with standard therapies should be recommended as preferred therapy for those patients with advanced pancreatic carcinoma. For vaccine-based immunotherapy, autologous activated lymphocyte therapy was considered to be the more promising and encouraging treatment compared with peptide-based vaccine therapy. Further clinical investigations, especially randomized controlled trials are warranted to determine the effectiveness and tolerability of immunotherapy.

## MATERIALS AND METHODS

### Trials identification

We conducted separate PubMed (inception to June 2016), EMBASE (inception to June 2016) and the Cochrane Library (inception to June 2016) searches of all relevant English language articles using an extension of the Cochrane search strategy [25]. Potential studies were identified by using the following keywords: “immunotherapy” OR “immunotherapeutic” OR “immune-cell based cancer therapy” OR “vaccine-based immunotherapy” AND “pancreatic cancer” OR “pancreatic carcinoma” OR “pancreatic adenocarcinoma” AND “advanced” OR “unresectable” OR “metastatic” OR “refractory” OR “inoperable”. Further information was manually searched reference lists from already retrieved studies and general medical journals (e.g., New England Journal of Medicine, The Lancet, British Journal of Medicine and JAMA) and journals in the cancer field (e.g., Journal of Clinical Oncology, Cancer Research, Cancer, Annals of Oncology, British Journal of Cancer, and Cancer Treatment Reviews). In particular, review articles were also examined for published results. By carefully checking the body of each publication and the name of all authors, we avoided duplications of data. The search strategy used is illustrated in Figure 1.

### Selection criteria

The selection criteria was as follows: (1) Trials were in the English language and were limited to human trials; (2) Prospective or retrospective clinical trials with or without a control population were eligible for inclusion; (3) Trials assessed the efficacy and safety of immunotherapy in the treatment of APC. (4) Trials reported endpoints without 95%CI or mean survival time were not included.

### Primary and secondary endpoints

Data on disease control rate (DCR) (partial response [PR] + complete response [CR] + stable disease [SD]), overall survival (OS), progression-free survival (PFS), 1-year survival rate and treatment-related adverse events (TRAEs) were independently extracted by two reviewer, with any discrepancies resolved by consensus (and if necessary with a third reviewer). The primary endpoint evaluated was DCR. The secondary endpoints evaluated were OS, PFS, 1-year survival rate and TRAEs. OS and PFS were estimated from the date of the initial treatment to the date of death or final follow-up and the date of disease progression, respectively. These endpoints were analyzed for all included studies for which data were available. The subgroup analyses were conducted according to the type of immunotherapy (autologous activated lymphocyte therapies, peptide vaccine therapy, and other immunotherapies such as monoclonal antibodies), combination with or without chemotherapy.

### Statistical analysis

We performed bootstrapping (number of samples = 1000) to calculate the 95%CI for OS, PFS, DCR and 1-year survival rate if study didn't report these endpoints’ 95%CIs but reported individual participant data. In particular, we used metaprop command (metaprop DCR [or 1-year survival rate], random second (fixed) ftt cimethod (exect) label (namevar = Author, yearvar = Year) to summarize DCR and 1-year survival rate. Considering the heterogeneity across studies, meta-analysis was performed using a random-effects model, using the DerSimonian-Laird method [26]. Heterogeneity across studies was assessed using the Cochran's Q and I2 statistic with values ranging from 0% (no heterogeneity) to 100%; values > 50% indicated significant heterogeneity [27]. Univariate meta-regression using REML method was performed to find out sources of heterogeneity including study design, mean age, female/male (F/M) ratio, number of patients, publication year, the type of immunotherapy, and combination with or without chemotherapy. Additionally, pre-specified subgroup analyses were performed (i) by type of immunotherapy; (ii) by combination with or without chemotherapy. Publication bias was examined utilizing funnel plots by the Begg's and Egger's methods. All data were recorded in a Microsoft Excel spreadsheet (Microsoft Corp, Redmond, WA) and analyzed using Statistical Package for Social Sciences (SPSS) software version 23.0 (SPSS Inc., Chicago, IL, USA) and STATA 12.0 (Stata Corp LP, College Station, Texas, USA).

## Abbreviations

APC: Advanced pancreatic cancer
DCR: Disease control rate
OS: Overall survival
PFS: Progression-free survival
TRAEs: Treatment-related adverse events
AALT: Autologous activated lymphocyte therapy
PBVT: peptide-based vaccine therapy
